# Characterization of HIV variants from paired Cerebrospinal fluid and Plasma samples in primary microglia and CD4^+^ T-cells

**DOI:** 10.1007/s13365-024-01207-w

**Published:** 2024-05-07

**Authors:** Stephanie B. H. Gumbs, Arjen J. Stam, Tania Mudrikova, Pauline J. Schipper, Andy I. M. Hoepelman, Petra M. van Ham, Anne L. Borst, LMarije Hofstra, Lavina Gharu, Stephanie van Wyk, Eduan Wilkinson, Lot D. de Witte, Annemarie M. J. Wensing, Monique Nijhuis

**Affiliations:** 1 Translational Virology, Department of Medical Microbiology, University Medical Center Utrecht, 3584 CX Utrecht, The Netherlands; 2 Department of Internal Medicine and Infectious Diseases, University Medical Center Utrecht, 3584 CX Utrecht, The Netherlands; 3 Centre for Epidemic Response and Innovation (CERI), School of Data Science and Computational Thinking, Stellenbosch University, Stellenbosch, South Africa; 4 KwaZulu-Natal Research Innovation and Sequencing Platform (KRISP), School of Laboratory Medicine and Medical Sciences, University of KwaZulu-Natal, Durban, South Africa; 5 Department of Psychiatry, Icahn School of Medicine at Mount Sinai, New York, NY 10029 USA

**Keywords:** HIV, CSF, Plasma, Compartmentalization, Microglia

## Abstract

Despite antiretroviral therapy (ART), HIV persistence in the central nervous system (CNS) continues to cause a range of cognitive impairments in people living with HIV (PLWH). Upon disease progression, transmigrating CCR5-using T-cell tropic viruses are hypothesized to evolve into macrophage-tropic viruses in the CNS that can efficiently infect low CD4-expressing cells, such as microglia. We examined HIV-1 RNA concentration, co-receptor usage, and CSF compartmentalization in paired CSF and blood samples from 19 adults not on treatment. Full-length envelope CSF- and plasma-derived reporter viruses were generated from 3 subjects and phenotypically characterized in human primary CD4^+^ T-cells and primary microglia. Median HIV RNA levels were higher in plasma than in CSF (5.01 vs. 4.12 log10 cp/mL; p = 0.004), and coreceptor usage was mostly concordant for CCR5 across the paired samples (n = 17). Genetically compartmentalized CSF viral populations were detected in 2 subjects, one with and one without neurological symptoms. All viral clones could replicate in T-cells (R5 T cell-tropic). In addition, 3 CSF and 1 plasma patient-derived viral clones also had the capacity to replicate in microglia/macrophages and, therefore have an intermediate macrophage tropic phenotype. Overall, with this study, we demonstrate that in a subset of PLWH, plasma-derived viruses undergo genetic and phenotypic evolution within the CNS, indicating viral infection and replication in CNS cells. It remains to be studied whether the intermediate macrophage-tropic phenotype observed in primary microglia represents a midpoint in the evolution towards a macrophage-tropic phenotype that can efficiently replicate in microglial cells and propagate viral infection in the CNS.

**Supplementary Information:**

The online version contains supplementary material available at 10.1007/s13365-024-01207-w.

## Introduction

Despite antiretroviral therapy (ART), HIV persistence in the central nervous system (CNS) continues to affect a large portion of people living with HIV (PLWH), resulting in a wide range of cognitive impairments (Heaton et al. 2010). The onset and progression of HIV-associated neurocognitive disorder (HAND) is believed to be multifactorial, including continued immune dysregulation and residual chronic inflammation in response to low-level virus production (or replication) and cytotoxic viral proteins (Clifford and Ances 2013; Jadhav and Nema 2021).

During early infection, R5 T-cell tropic viruses, characterized by their ability to efficiently enter CD4^+^ T-cells but not macrophages and microglia, represent the majority of the viral population (Joseph and Swanstrom 2018). Upon disease progression, genetically distinct viral populations can be found in cerebrospinal fluid (CSF) and brain tissue of both untreated and virally suppressed individuals, irrespective of the presence of neurological disorders (Bednar et al. 2015; Borrajo et al. 2021; Chan and Spudich 2022). Extensive research has been conducted on the genetic compartmentalization between the CNS and blood, however their phenotypic characteristics remain poorly understood. R5 T-cell tropic viruses are predominant in both compartments. In general R5 plasma derived viruses are T cell tropic, while CNS derived viruses, in addition to T cell tropic virus, can also harbor viruses that are M-tropic, referring to their enhanced ability to infect cells with low CD4 surface expression such as macrophages and microglia (Brese et al. 2018; Gonzalez-Perez et al. 2012; Schnell et al. 2011; Sturdevant et al. 2012). Accordingly, HIV DNA and/or RNA within the CNS are mostly found in perivascular macrophages and microglia (Ko et al. 2019; Lamers et al. 2016; Tso et al. 2018).

Previous studies have examined the CD4 entry phenotype of CNS- and plasma-derived pseudotyped viruses using the Affinofile cell line, on which CD4 and CCR5 surface expression can be differentially induced, and monocyte-derived macrophages (Arrildt et al. 2015; Joseph et al. 2014; Schnell et al. 2011). While the Affinofile cell line is commonly used for entry tropism analysis, this model system is derived from a T-cell line and therefore cannot fully represent the entry determinants for primary microglia, such as attachment receptors, endocytosis mechanisms, and microglia-specific restriction factors. Therefore, it remains to be determined whether M-tropic HIV variants have the same entry advantage for microglia as they do for low CD4 Affinofile cells and monocyte-derived macrophages. In this study, we examined potential genetic compartmentalization between paired CSF- and plasma-derived HIV variants and gain more insight into their entry affinity for human primary CD4^+^ T-cells and primary microglia. Paired CSF- and plasma-derived HIV variants were isolated from viremic PLWH without antiretroviral treatment and characterized based on coreceptor-usage and genetic compartmentalization, followed by a phenotypical analysis in CD4^+^ T-cells and microglia. To our knowledge, this is the first study to combine genetic characterization with a phenotypical analysis in human primary blood and CNS cells.

## Methods

### Design and study population

For this cross-sectional study, paired CSF-plasma samples were collected from stored samples in the period 2001–2016 from patients in care at the University Medical Center Utrecht and participating in the Dutch ATHENA observational cohort. Paired samples were obtained for clinical diagnostic purposes [Table S1]. A total of 28 subjects, with and without neurological symptoms, had sufficient material for virological analysis, of which 9 were excluded due to being on different antiretroviral therapies at the time of sampling. CSF in neurosymptomatic subjects was collected for clinical diagnostics purposes. The CSF of neuroasymptomatic patients was primarily collected to exclude neurosyphilis, as part of a standard clinical procedure in patients with TPHA (treponema pallidum hemagglutination assay) or (previous) VDRL- positive plasma syphilis serology and in two instances for a diagnostic work-up not including neurological symptoms [S1]. Paired samples were defined as CSF and plasma-EDTA (or serum) obtained within 7 days from each other. All patients had detectable HIV RNA in plasma at the time of lumbar puncture and were ART-naïve or off-treatment at the time of sampling [Tables 1 and S1].

**Table 1:** Clinical, virologic and phylogenetic characteristics of the subject population

								**Analysis of compartmentalization**^**g**^			
**Subject ID**	**Disease state (CDC)**^**a**^	**CD4**^**b**^	**NS/NA**^**c**^	**Origin**	**HIV VL**^**d**^	**FPR Range**^**e**^	**Tropism**^**f**^	**Fst**	**Snn**	**SM**	**CSF Compart.**^**h**^
1	A2	209	NA	CSF	3.95	30.1—73.3	R5	0.20	0.02	0.302	EQ
				Plasma	5.65	30.1—37.1	R5				
2	A2	373	NA	CSF	3.21	17.3	R5	N/A	N/A	N/A	N/A
				Plasma	5.01	17.3—46.8	R5				
3	unknown	549	NA	CSF	4.72	76.2—95.2	R5	0.66	0.91	1	EQ
				Plasma	5.88	68.6—95.2	R5				
4	unknown	400	NA	CSF	3.13	12	R5	N/A	N/A	N/A	N/A
				Plasma	3.15	12—33.7	R5				
6	C3	16	NS	CSF	4.88	100	R5	0.15	< 0.0001	0.09	EQ
				Plasma	5.34	4.8—99.2	R5				
7	B2	246	NA	CSF	3.72	26.2—78.1	R5	0.70	0.38	0.33	EQ
				Plasma	4.31	48.7—72.1	R5				
8	unknown	10	NS	CSF	5.28	7.8—44.2	R5	0.33	0.67	0.26	EQ
				Plasma	5.35	4—81	R5				
10	A1	705	NA	CSF	4.43	83—97	R5	0.84	0.80	0.24	EQ
				Plasma	5.10	83—97	R5				
12	A2	375	NA	CSF	4.72	6.3—20.4	R5	0.21	0.59	0.1	EQ
				Plasma	4.49	10.5—20.4	R5				
13	unknown	30	NS	CSF	5.21	74.6—91.2	R5	< 0.0001	< 0.0001	0.01	CP
				Plasma	6.04	35.1—53.7	R5				
14	A2	469	NS	CSF	3.54	71.1	R5	0.54	0.34	0.59	EQ
				Plasma	4.27	71.1—90.7	R5				
16	C3	83	NS	CSF	4.12	72—94.6	R5	0.72	0.86	0.6	EQ
				Plasma	5.10	76—94.6	R5				
17	A1	424	NS	CSF	4.38	2.5—5	X4/R5	0.70	0.01	0.04	EQ
				Plasma	3.17	1.7—55.1	X4/R5				
18	A1	574	NS	CSF	2.87	15—46.8	R5	0.44	0.33	0.13	EQ
				Plasma	4.56	15—46.8	R5				
19	A1	387	NA	CSF	4.77	16—87	R5	< 0.0001	< 0.0001	< 0.0001	CP
				Plasma	4.18	0.2—90.3	X4/R5				
20	A0	139	NA	CSF	3.25	64—70.8	R5	0.27	0.53	1	EQ
				Plasma	3.71	56.1—74.4	R5				
21	A2	399	NS	CSF	3.74	30.1—52.1	R5	0.29	0.92	0.16	EQ
				Plasma	3.78	30.1—52.1	R5				
25	A0	362	NS	CSF	3.06	25.2	R5	N/A	N/A	N/A	N/A
				Plasma	5.88	25.2	R5				
27	C3	185	NS	CSF	4.95	38.8—86.2	R5	0.56	0.73	0.38	EQ
				Plasma	5.73	8.5—86.2	R5				

N/A.: V3 sequences clonal in CSF or identical between CSF and plasma

^a^HIV disease stage according to the CDC 1993 Revised Classification System for HIV Infection (PMID: 1361652)

^b^estimated plasma CD4 cells/μL, value determined by test value closest to sampling time of pair

^c^*NS* neurosymptomatic, *NA* neuroasymptomatic

^d^*VL* viral load; estimated HIV-RNA (log10 copies/ml) determined by test value closest to sampling time of pair

^e^*FPR* False-positive rate; lowest and highest FPR detected in CSF and plasma based on V3 amplicons (Miseq sequencing) with the geno2pheno algorithm

^f^Geno2pheno coreceptor prediction; R5: virus predicted to use the CCR5 co-receptor. X4: virus predicted to use the CXCR4 co-receptor

^g^Comparative genetic analyses of viral populations in blood plasma and cerebrospinal fluid using three statistical analyses: Wright’s measure of population subdivision (Fst), Nearest-neighbor statistic (Snn) and the Slatkin-Maddison test (SM). Genetic compartmentalization was statistically significant at P values < 0.01

^h^Characteristics of the HIV viral population in the CSF compartment (compart): CP (compartmentalized), if all three tests were significant (p < 0.01), or equilibrated (EQ) if statistical significance was not reached (p > 0.01)

### HIV RNA and plasma CD4 count analysis

HIV RNA levels in plasma and CSF were determined by an ultrasensitive viral load assay with a reported cut-off value of 50 copies/ml (Ampliprep/COBAS Taqman HIV-1 assay, Roche).

### Next-generation sequencing and co-receptor prediction

Viral RNA was isolated according to the method developed by (Boom et al. 1990). The HIV-1 envelope V3 region was amplified by RT-PCR (Titan One Tube RT-PCR kit, Roche) followed by a nested PCR (Expand High Fidelity PCR System, Roche), according to the manufacturer’s protocol. Please refer to supplementary Table S2 for a list of the primers used. PCR products were purified with the Qiaquick PCR purification kit (Qiagen). Library preparation was done using a Nextera-XT DNA Library Preparation and Index kit (Illumina, USA) according to the manufacturer’s instructions. The resulting libraries were normalized and pooled. Sequencing was performed on an Illumina MiSeq platform using the MiSeq Reagent Kit v2 for 500 cycles. After aligning the sequence reads of each subject with the consensus sequence of their respective subtype, reads that overlap the entire V3 region were isolated and trimmed. Unique V3 sequences with a prevalence of > 1% in the population were used for HIV-1 co-receptor tropism. Co-receptor usage was predicted with the Geno2pheno[coreceptor] algorithm version 2.5 for deep sequences with the recommended false-positive rate (FPR) cut-off value for deep V3 sequencing of 3.5% (Swenson et al. 2011). It is predicted that an FPR value below 3.5% indicates an X4-tropic virus, whereas a value above 3.5% indicates an R5-tropic virus.

### Compartmentalization analysis

Genetic compartmentalization between the CSF and plasma-derived viral population, was determined for each subject based on only deep-sequenced V3 sequences using three methods: Wright’s measure of population subdivision (Fst) (Hudson et al. 1992), Nearest-neighbor statistic (Snn) (Hudson 2000), and the tree-based Slatkin-Maddison test (SM) (Slatkin and Maddison 1989). Wright’s measure of population subdivision (Fst) quantifies the genetic variance between populations relative to the total genetic variance. Higher Fst values indicate greater population differentiation. The nearest-neighbor statistic (Snn) assesses how often sequences' nearest neighbors are from the same compartment, with values closer to 1 indicating stronger compartmentalization. The Slatkin-Maddison (SM) test evaluates the number of migrations needed to explain the distribution of lineages between populations, with fewer migrations suggesting compartmentalization. All three methods were conducted with the HyPhy software version 2.2.4 (Kosakovsky Pond et al. 2005). For the two distance-based methods (Fst and Snn), the Tamura-Nei 93 algorithm was applied along with a bootstrap value and permutation test of 10,000 with only deep-sequenced V3 sequences as input. For the tree-based SM test, multiple sequence alignments of the HIV *env* V3 regions were performed using the online version of the MAFFT software (https://mafft.cbrc.jp/alignment/server/index.html) (Katoh et al. 2018), accessed 2022/08/09 using standard parameters for nucleic acid alignment. A Nearest Neighbor Joining Tree was constructed using MEGA version 11.0.11(Tamura et al. 2021), and the Kimura 2-parameter substitution model was applied. Compartmentalization was evaluated using the nearest-neighbor statistic (Snn) (Hudson 2000), applying 10 000 permutations, implemented using the HyPhy software package version 2.2.4 (Kosakovsky Pond et al. 2005). CSF viral populations were defined as either compartmentalized (cp), if all three tests were significant (p < 0.01), or equilibrated (eq) if statistical significance was not reached (p > 0.01) in one or more of these tests (Zárate et al. 2007).

### Generation of HIV viral clones

The HIV-1 envelope (gp160) region was amplified by RT-PCR (Superscript IV Reverse Transcriptase Kit, Invitrogen) followed by a nested PCR (Platinum Taq Superfi PCR Master Mix, Invitrogen), according to the manufacturer’s protocol. Please refer to supplementary Table S2 for a list of the primers used. Envelope amplicons were introduced into a HxB2 gp160deletion vector with a luciferase reporter gene (HxB2ΔENVluc), previously described in (Gumbs et al. 2022), using the NEBuilder HiFi DNA Assembly Cloning Kit (New England Biolabs). We used the same vector carrying either the gp160 sequence of JRCSF (R5 T-tropic), YU-2 (R5 M-tropic) or BaL (R5-tropic) as controls. Hek-293 T cells were transfected with the chimeric plasmids using lipofectamine 2000 reagent (Invitrogen). After 48 h, the supernatant containing replication-competent virus was harvested and stored at −80 °C until further use. p24 was determined with an ELISA p24 assay (Aalto Bioreagent, Dublin, Ireland).

### HIV Infection in primary microglia and CD4^+^ T-cells

Fresh postmortem adult human brain tissue was provided by the Netherlands Brain Bank (NBB). The isolation of primary microglia was conducted according to the protocol described previously with some minor modifications for human brain tissue (Mattei et al. 2020). Following isolation, primary microglia were cultured in poly-L-lysine hydrobromide (PLL)-coated 96-well plates (1 × 10^5^ cells/well) in microglia medium (RPMI 1640 (Gibco Life Technologies) supplemented with 10% FCS, 1% penicillin–streptomycin (Gibco Life Technologies) and 100 ng/mL IL-34 (Miltenyi Biotec)) for 2–3 days before infection. Primary microglia were infected overnight with 10 ng (p24Gag) virus after which the medium was fully replaced and cells were cultured up to 17 days in microglia medium without medium refreshment.

PBMC were isolated from peripheral blood obtained from healthy donors by Ficoll-Paque density gradient. CD4^+^ T-cells were subsequently isolated through negative selection with the CD4^+^ T Cell Isolation Kit (Miltenyi Biotec 130–096-533), according to the manufacturer’s protocol. Before infection, CD4^+^ T-cells were stimulated for 2 days in culture medium (RPMI 1640 (Gibco Life Technologies) with 10% Fetal Bovine Serum, 1% penicillin–streptomycin (Gibco Life Technologies) and IL-2 (20U/mL) (Invitrogen)) supplemented with Phytohaemagglutinin (5 µg/mL). CD4 infection was performed in Duplo for 3 h with 10 ng (p24 Gag) virus per 100.000 cells in Eppendorf’s placed on a tube rotator. For the Maraviroc experiment, CD4^+^ T-cells were treated with 100 nM MVC for 1 h before infection. Following infection, medium was fully replaced with culture medium and CD4^+^ T-cells were cultured for 14 days in 96-well plates without medium refreshment.

### Luminescence

Supernatant was collected 2–3 times per week and luminescence was measured according to the manufacturer’s protocol with the Nano-Glo® Luciferase Assay System (Promega). The graphs were created with GraphPad Prism version 8.3.0.

### Statistical analysis

All data were analyzed with GraphPad Prism version 8.3.0. Descriptive statistics were used to compare the characteristics between the paired plasma and CSF samples. The nonparametric Wilcoxon signed rank test is used to compare groups with paired data, including the differences in FPR. Differences within continuous variables (e.g., viral load) compared to categorical data (e.g., neurological symptoms) were performed by using the Mann–Whitney U test. A Pearson correlation is used to determine the association of HIV-RNA levels.

## Results

### Clinical characteristics

Paired plasma and CSF samples were collected from 19 adult subjects enrolled in the Dutch ATHENA observational cohort subjects were mainly infected with Subtype B virus, except for subject 6 (subtype CRF02_AG) and subject 25 (CRF12_BF), and were ART naïve or off treatment at the time of sampling for at least 1 month. The majority of the study population was male, with a mean age of 47 years and a mean CD4^+^ T-cell count of 312 cells/µL. The clinical and virological characteristics of each subject can be found in Table 1 and Table S1.

**Fig. 1:**
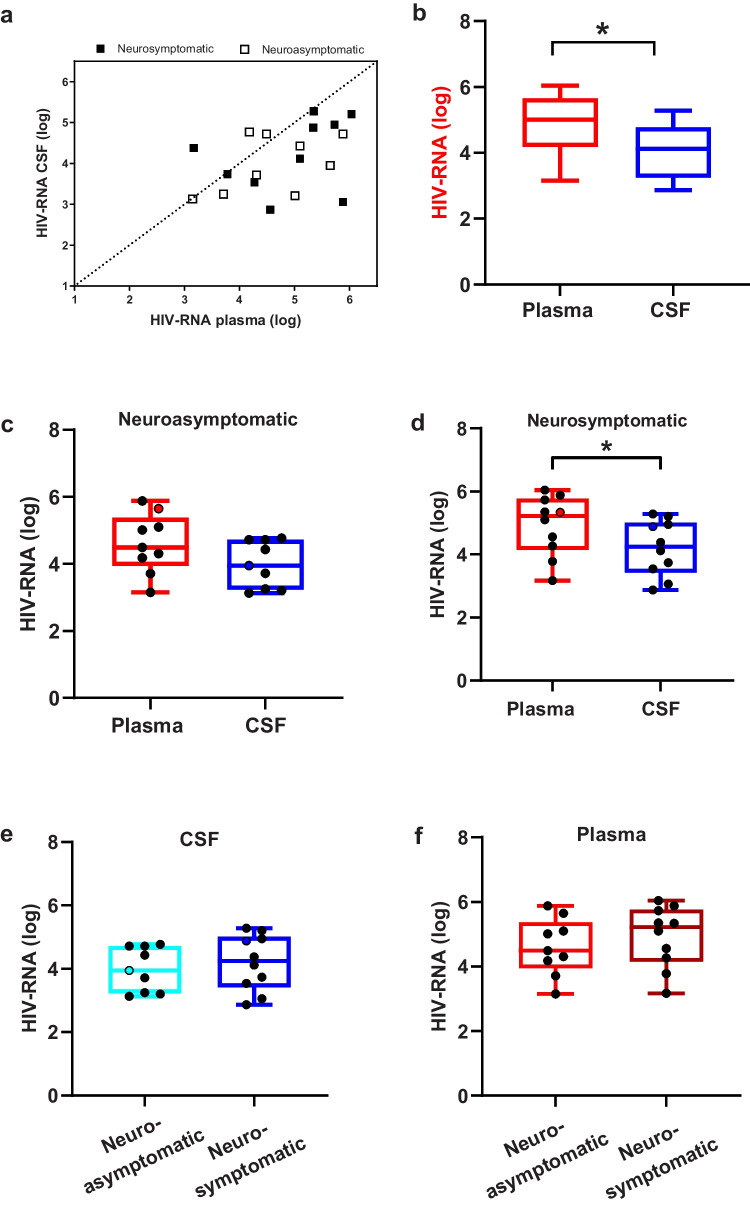
Relationship of HIV-1 RNA levels (log 10cp/mL) measured in paired plasma and CSF samples in neuroasymptomatic and neurosymptomatic subjects. **a** Plots depicts the correlation between HIV RNA CSF and HIV RNA plasma for all subjects. Black dashed line represents line of identity. **b, c, d** Boxes depict median HIV RNA levels and IQR, measured in plasma (red) and CSF (blue) in all patients, neuroasymptomatic and neurosymptomatic subjects. **e, f** Boxes depict CSF (blue) and plasma (red) median HIV RNA levels and IQR between neuroasymptomatic and neurosymptomatic subjects. * = Statistically significant (p < 0.05)

Median HIV RNA levels were significantly higher in plasma than in CSF (5.01 vs. 4.12 log10 cp/mL; p = 0.004) and moderately correlated with each other (Pearson r = 0.42; p = 0.04) [Fig. 1a, b]. A similar pattern in virus concentration was also observed in the neurosymptomatic subjects (5.22 vs. 4.25 log10 cp/mL; p = 0.05) [Fig. 1d]. However, further research with a larger sample size is needed to determine whether this finding is also true for neuroasymptomatic subjects [Fig. 1c]. A comparison of the CSF and plasma RNA levels between the neurosymptomatic and neuroasymptomatic subjects, however, showed no significant difference [Fig. 1e, f].

### Analyses of CNS compartmentalization

Genetic compartmentalization between CSF and plasma HIV variants was determined based on the V3 region in the viral envelope (env) gene. CSF viral populations were defined as either compartmentalized (cp), if all three compartmentalization analyses (Fst, Snn, SM) were significant (p < 0.01), or equilibrated (eq) if statistical significance was not reached (p > 0.01) (Zárate et al. 2007) [Table 1]. Most of the subjects (89%) had equilibrated viral populations in their CSF and plasma. Significant genetic CNS compartmentalization was detected in two subjects, 13 and 19. Subject 13 had advanced disease (CD4 count 30 cells/μl) and neurological symptoms consisting of balance disturbances and peripheral weakness. In contrast, subject 19 had less advanced HIV infection (CD4 count 387 cells/μl) and had no neurological symptoms, suggesting that CNS compartmentalization does not always present with clinically observable neurological symptoms.

### CSF-derived viral variants can efficiently enter CD4^+^ T-cells, with a modest enhancement for viral entry in low-CD4 expressing primary microglia

Co-receptor usage was mostly concordant across the paired samples, with 17 out of 19 pairs predicted to consist exclusively of CCR5-using viral strains in both compartments [Table 1]. A comparison of the lowest FPR values in plasma and CSF revealed no correlation or significant difference [Data not shown]. Subject 17 is predicted to have CCR5- and CXCR4-using viral strains in both plasma and CSF, whereas subject 19 is predicted to have CXCR4- and CCR5-using viruses in the plasma but only CCR5-using virus in the CSF. In addition to being the only subject with CXCR4-using virus in the CSF, subject 17 was also the only subject diagnosed with severe symptoms (HIV encephalopathy) and one of three subjects with a CSF HIV RNA load higher than in plasma (difference 1.21 log10 copies/mL).

**Fig. 2:**
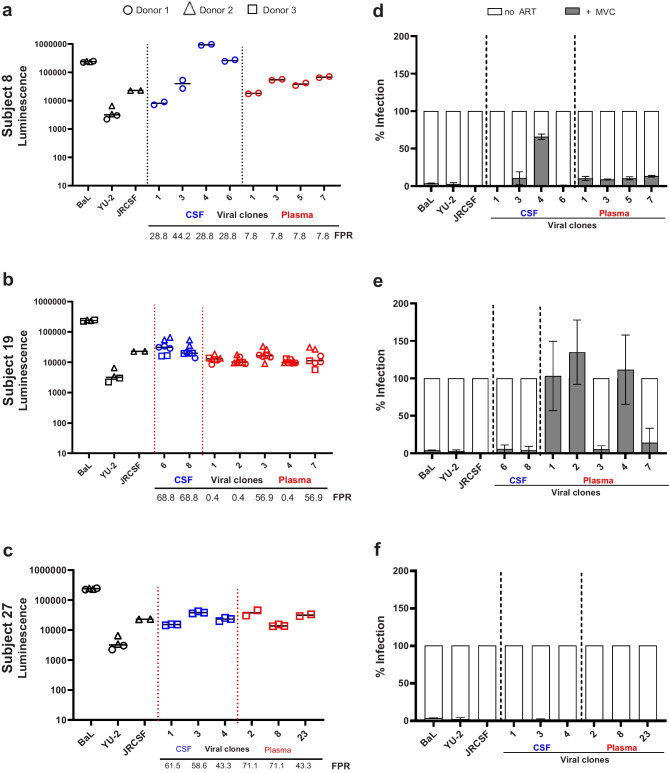
CSF- and plasma-derived viruses can efficiently infect and replicate in CD4^+^ T-cells. **a-c** CD4^+^ T-cells were infected with 10 ng (p24 Gag) CSF- or plasma-derived virus generated from subjects 8, 19 and 27. Scattered dot plots depict luciferase activity measured in supernatant collected on day 14 post-infection, whereas horizontal black lines represent median luminescence. FPR values for each clone are included at the bottom of the figure. An FPR value below 3.5% is predicted to be X4-tropic virus. An FPR value above 3.5% is predicted to be R5-tropic virus. **(d-f)** CD4^+^ T-cells were untreated (no ART) or treated with 100 nM Maraviroc (MVC) prior to infection with 10 ng (p24 Gag) CSF- or plasma-derived virus generated from subjects 8, 19 and 27. Bar graphs depict the maximum infection measured on day 14 post-infection with (grey) and without (white) treatment with MVC. Black error bars depict standard error of the means. Lab strains and viruses derived from plasma (red) or CSF (blue) are separated by vertical dotted lines

Within the CNS, HIV DNA is primarily detected in perivascular macrophages and primary microglia (Joseph et al. 2015). We investigated the entry phenotype of the CSF and plasma HIV variants from compartmentalized (cp) neuroasymptomatic subject 19 and two equilibrated (eq) subjects, subjects 8 and 27, with neurological symptoms [Table S1]. These three subjects were selected in a step-wise process. First, we selected subjects with a difference in FPR value between plasma and CSF, then we selected subjects from whom the volume of stored CSF and plasma was sufficient for the experiment and finally the clones needed to be viable from both plasma and CSF. First, we generated CSF- and plasma-derived luciferase reporter viruses using the full-length envelope (*Env*) gene amplified from the CSF and the plasma of these three subjects. For each subject, we obtained a diverse mixture of viral clones with different FPR values between the CSF and plasma [Fig. 2]. These viral clones were phenotypically characterized for viral entry into CD4^+^ T-cells (high CD4 surface levels) and primary microglia (low CD4 surface levels), the main HIV target cells in the blood and CNS. Considering that CD4 surface expression levels on CD4^+^ T-cells and microglia are likely to differ between donors, we used 3 different donors and three laboratory strains as a control for infection: two R5 M-tropic virus (Bal and YU-2) and one R5 T-tropic virus (JRCSF).

For subjects 19 (cp) and 27 (eq), we observed no significant difference between the ability of the CSF and plasma viruses to infect CD4^+^ T-cells [Fig. 2b, c]. In subject 8 (eq), we observed a ≥ 10-fold higher infectivity with CSF clones 4 and 6, compared to the plasma viruses [Fig. 2a]. Interestingly, this infectivity was also substantially higher than CSF clone 1 which had the same FPR value, suggesting that there are other determinants outside of the V3 loop that can greatly affect cell entry. Furthermore, treatment with the CCR5 inhibitor maraviroc (MVC), supported co-receptor prediction and significantly inhibited T-cell infection by the R5-predicted CSF and plasma viruses, whereas the X4-predicted plasma viruses of subject 19 were resistant to MVC inhibition [Fig. 2d–f]. R5-predicted CSF clone 4 of subject 8, despite having a high FPR value of 28.8, was also greatly resistant to MVC inhibition (≥ 60%), suggesting that this clone is dual tropic [Fig. 2d].

**Fig. 3:**
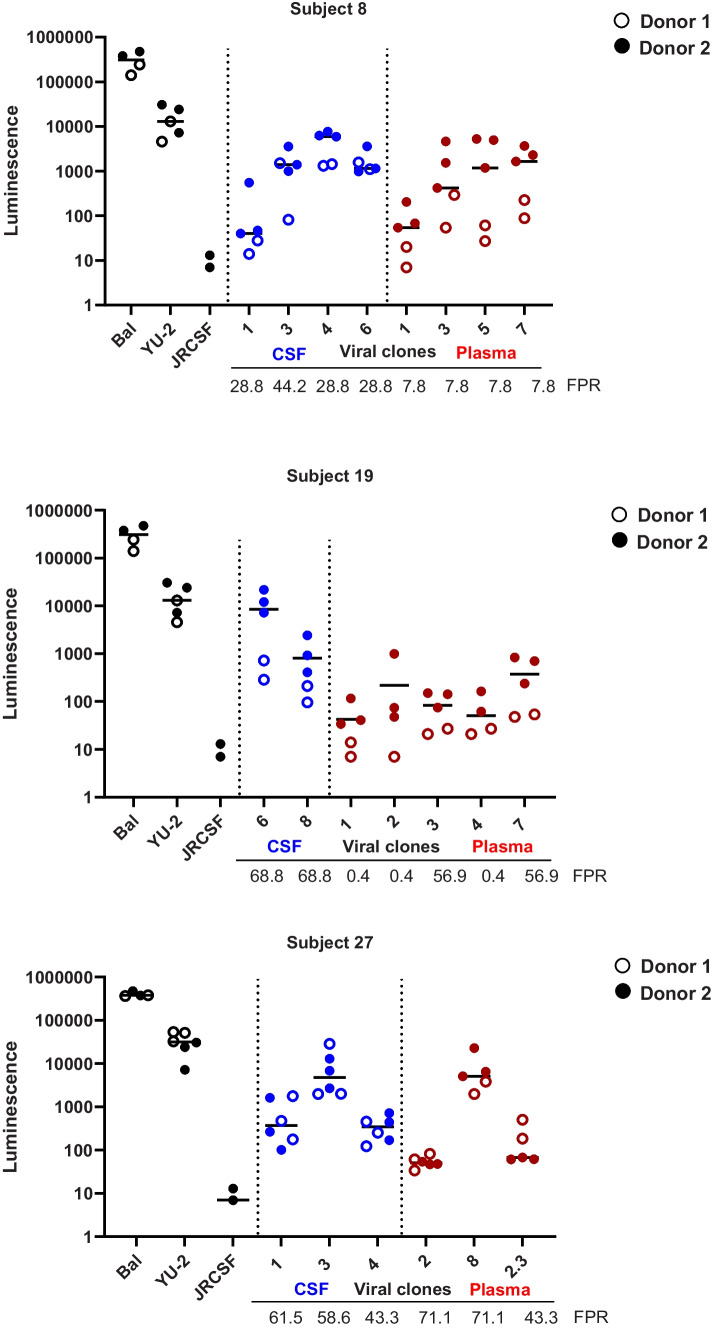
CSF-derived viruses have a modest enhancement for infecting primary microglia. **a-c** Primary microglia were infected with 10 ng (p24 Gag) virus generated from subject 8, 19 and 27. Graphs represent luciferase activity measured in supernatant collected on Day 17 post-infection. Black line depicts the median luminescence measured. Lab strains and viruses derived from plasma (red) or CSF (blue) are separated by vertical dotted lines

**Fig. 4:**
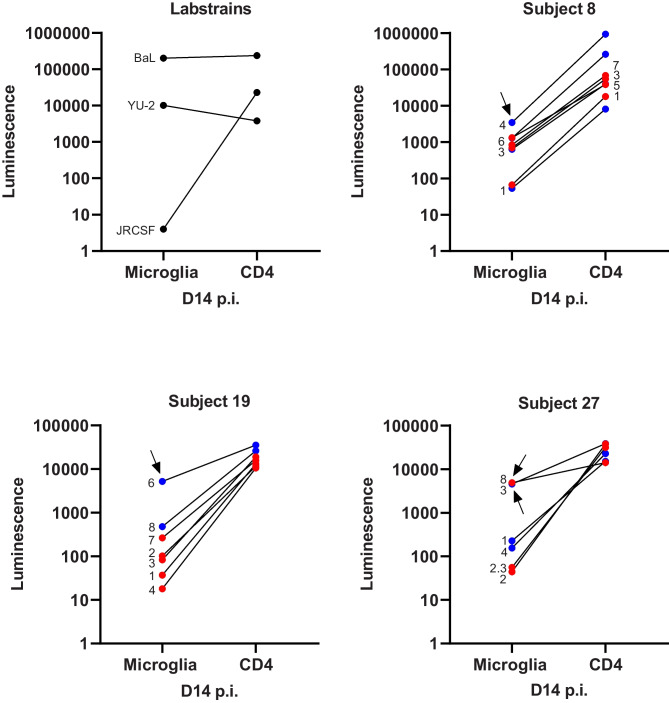
Majority of the CSF and plasma viral population displayed the typical R5 T-cell tropic phenotype. Graphs represent luciferase activity measured in supernatant collected on Day 14 post-infection (D14 p.i.) in primary microglia and CD4^+^ T-cells. Laboratory strains are depicted in black, CSF-derived clones in blue and plasma-derived clones in red. Black arrows indicate viral clones with an intermediate M-tropic phenotype

Lastly, phenotyping of the viruses in low CD4-expressing primary microglia revealed that most CSF and plasma clones were unable to efficiently enter these cells, although CSF-derived viral clones were overall better in entering microglial cells than the plasma-derived clones [Fig. 3]. The enhanced ability of CSF clones, to infect microglia was more pronounced in compartmentalized subject 19 [Fig. 3b]. While both BaL and YU-2 are R5 M-tropic viruses, BaL was isolated from infant lung tissue (Gartner et al. 1986), whereas YU-2 was isolated from brain tissue (Li et al. 1991) and hereby potentially more representative of the neurotropic viruses in the CNS. From this perspective, we compared the infection of the viral clones to YU-2 and found that each subject had one CSF clone with an intermediate M-tropic phenotype, defined as ≥ 50% of YU-2 infection, for cell entry in microglia [Fig. 4]. In subject 27, we also found one plasma clone with this intermediate M-tropic entry phenotype. The plasma clone had a higher FPR than the CSF clone with a similar intermediate phenotype (71.1 vs. 58.6), suggesting two separate virus populations possibly originating from different low CD4- expressing cells.

## Discussion

With up to 43% of the HIV-infected population still affected by lasting HIV-associated neurological impairments despite viral suppression with ART, research on the neuropathogenesis of HIV remains essential (Wang et al. 2020). In this study, we report, for the first time, that patient-derived replication-competent HIV variants can infect and replicate in human primary microglia, however, HIV replication was more efficient in primary CD4^+^ T-cells.

HIV RNA can be detected in the CSF as early as 8 days post-estimated infection, however, RNA levels in CSF are generally lower than in plasma (Valcour et al. 2012). Within our study population, HIV RNA levels were significantly lower in CSF than in plasma, however, 16% (n = 3) of the subjects (subjects 12, 17, 19) had higher virus concentration in CSF than in plasma, a phenomenon associated with HAND (Bai et al. 2017). Among these three subjects, only subject 17 had neurological symptoms at the time of sampling (HIV encephalopathy). A recent multicenter study reported that up to 30% of treatment-naïve individuals with HIV-associated dementia (HAD) had CSF to plasma HIV RNA discordance (Ulfhammer et al. 2022). The detection of higher levels of HIV RNA in CSF than in plasma suggests compartmentalized viral production and/or replication in CNS resident cells.

Compartmentalization is observed in some but not all subjects. We found a genetically compartmentalized viral population in the CSF in 2 subjects (subjects 13 and 19). It is thought that within the first two years of infection, CSF compartmentalized variants are predominantly R5 T-tropic and associated with clonal amplification and the presence of elevated CSF pleocytosis (Sturdevant et al. 2015). HIV infection may progress in advanced stages of disease to HAD, in which both compartmentalized R5 T-tropic and R5 M-tropic CSF viral populations can be detected in the CSF (Schnell et al. 2011). Based on genetic analysis, these R5 M-tropic viruses are more genetically diverse than the R5 T-tropic viruses, which suggests that they are replicating in the long-lived cells of the CNS (Arrildt et al. 2015; Schnell et al. 2011). In our study, subjects 13 and 19 both had compartmentalized R5-using CSF viruses, however, only subject 13 had reported neurological symptoms at the time of sampling, suggesting distinct viral tropism between the subjects, namely R5 M-tropic (subject 13) and R5 T-tropic (subject 19). In addition, an X4-using viral population was found in the CSF of subject 17 who was diagnosed with HIV encephalopathy. As the only subject with severe neurological symptoms, it remains to be determined whether the prevalence of X4-using virus in the CNS is associated with the progression of neurological disease.

In the CNS, HIV is primarily detected in perivascular macrophages and primary microglia that both express the CCR5 co-receptor (Joseph et al. 2015). In this study, we phenotypically characterized CSF- and plasma-derived viral clones from compartmentalized subjects 19 and equilibrated subjects 8 and 27 for their ability to infect and replicate in CD4^+^ T-cells and primary microglia. Characteristic of both M- and T-tropic viruses, all viral clones were able to effectively infect high CD4-expressing T-cells with no major differences between the CSF and the plasma viruses. Treatment with MVC confirmed productive infection and corroborated the prediction of both X4 and R5-using viruses in the plasma of subject 19 and revealed a possible dual-tropic viral population in subject 8. Interestingly, we also observed an enhanced infection of the X4-using viruses following treatment with MVC, suggesting that treatment of CD4^+^ T-cells with MVC increases their susceptibility to X4-using viral infection possibly due to cell activation (López-Huertas et al. 2020; Madrid-Elena et al. 2018).

Furthermore, In line with previous studies on monocyte-derived macrophages and Affinofile cells (Brese et al. 2018; Gonzalez-Perez et al. 2012; Schnell et al. 2011; Sturdevant et al. 2012), CSF-derived viral clones were overall more efficient at infecting low CD4-expressing primary microglia than the plasma-derived clones, despite differences among donors. The infection levels of the CSF-derived clones, however, never reached the level of the R5 M-tropic laboratory strains Bal and YU-2, and therefore did not meet the criteria for an M-tropic phenotype. R5 T-cell tropic viruses found in the CSF are presumed to originate from infiltrating infected CD4^+^ T-cells or are potentially produced by resident CD4^+^ T-cells in the brain parenchyma (Joseph and Swanstrom 2018; Schnell et al. 2011). Nonetheless, we observed an intermediate M-tropic phenotype, defined as ≥ 50% of YU-2 infection, for several CSF clones (one in each subject) and one plasma clone. Other than CSF and plasma (Arrildt et al. 2015; Joseph et al. 2014; Sturdevant et al. 2015), viruses with an intermediate M-tropic phenotype, determined by low CD4 Affinofile cells and/or monocyte-derived macrophages, have also been detected in peripheral tissues, such as the colon, lungs, and lymph nodes (Brese et al. 2018). Due to the relatively invasive nature of CSF collection, longitudinal samples were not obtained, therefore we were not able to determine whether this intermediate M-tropic phenotype represents an evolutionary intermediate on the path to macrophage tropism. A recent paper by Woodburn et al. reported that patient-derived M-tropic HIV Env proteins confer an entry advantage over T cell-tropic Envs when infecting primary microglia (Woodburn et al. 2022). In line with our study, infection of primary microglia with an R5 T-cell tropic virus with an intermediate M-tropic phenotype approached but did not reach the infection level of the M-tropic viruses.

It is hypothesized that the enhanced ability of M-tropic viruses to utilize low CD4 surface expression for viral entry is marked by an increased Env: CD4 affinity, enhanced sensitivity to sCD4 inhibition, and other subtle changes in the trimer conformation of the Env protein. Several studies have reported a variety of substitutions in the envelope gene found to be associated with M-tropic CNS-derived viruses, such as N283 in the CD4 binding site (CD4bs) (Dunfee et al. 2006), a conserved amino acid in the V1 loop (Musich et al. 2011), and the loss of an N-linked glycosylation site at 386 (Duenas-Decamp et al. 2009; Dunfee et al. 2007). However, none of these genetic mutations were conserved across different studies. Interestingly, non-M-tropic viruses were recently shown to productively and efficiently infect macrophages through Env-dependent cell–cell fusion with infected CD4^+^ T-cells (Han et al. 2022). The Envs expressed on infected T-cells also showed enhanced interaction with the CD4 and CCR5 receptors and were less dependent on the surface density, compared to the cell-free virus-associated Envs. However, the infection of microglial cells in vivo through cell-to-cell fusion with infiltrating infected CD4^+^ T-cells is yet to be demonstrated. In addition, the limited infection of primary microglia observed with both M- and T-tropic viruses can also be attributed to the host-restriction factors expressed in microglia, such as Sp3 protein and C-EBPγ, that function as transcriptional repressors (Wallet et al. 2019).

Furthermore, we recognize that our study has several limitations. First, this study, utilized plasma and CSF samples obtained for clinical diagnosis, which limited the number of participants that could be included. Second, in this small study sample, we were not able to establish a relation between clinical symptoms and CNS diversification. Third, we used the Env protein to generate recombinant viral clones rather than using full-length viral clones. While the envelope protein is the major determinant for co-receptor usage and CD4 binding, we cannot completely rule out the possibility of viral evolution outside of the Env gene that contributes to the M-tropic phenotype. Finally, we used a cell-free virus infection assay which might not fully represent the modes of microglia infection in vivo*.*

Nonetheless, we were able to derive significant and compelling evidence that supports the CNS as a viral reservoir for HIV in a subset of patients. Among these findings is the detection of a genetically distinct CSF viral population, indicating viral replication in the CNS. In addition, we detected CSF-derived viral clones that exhibit a modestly enhanced ability to enter primary microglia. Ultimately, the evidence of viral replication and evolution in the CNS highlights the importance of the CNS as a HIV reservoir.

## Supplementary Information

Below is the link to the electronic supplementary material.

Supplementary file1 (PDF 155 KB)

Supplementary file2 (PDF 80 KB)

## Data Availability

The data that support the findings of this study are available from the corresponding author upon reasonable request.
