# Supplementary material for: Characterization of HIV variants from paired Cerebrospinal fluid and Plasma samples in primary microglia and CD4+ T-cells
Source: J Neurovirol. 2024 May 7;30(4):380–92. doi: 10.1007/s13365-024-01207-w (PMC11512886; doi:10.1007/s13365-024-01207-w)
Supplement: Supplementary file 1 — Supplementary file1 (PDF 155 KB) [file 13365_2024_1207_MOESM1_ESM.pdf]

Supplementary table 1: Neurological diagnosis and ART history of subject population

Subject ID	Subtype	Indication for CSF puncture [a]	Serum THPA	Serum VDRL	CSF THPA	CSF VDRL	Neurological symptoms [b]	ART history	Time off-treatment [c]	Year of diagnosis
1	B	Exclusion NS	640/pos	4/neg	4/pos	<4/neg	-	-		2006
2	B	Exclusion NS	-	<4/neg		<4/neg	-	-		2006
3	B	Exclusion NS	>2560/pos	32/pos	512/pos	<4/neg	-	-		2003
4	B	Symptoms, other than neurological	<80/neg	-	<4/neg	-	-	3TC, ZDV, RTV, d4T, SQV, ddI	>1 year	1992
6	CRF02_AG	Symptoms	<80/neg	-	-	-	Dizzyness and confusion	-		2003
7	B	Exclusion NS	>2560/pos	320/pos	8/pos	<4/neg	-	-		2004
8	B	Symptoms	1280/pos	<4/neg	-	-	Cryptococcal meningitis	-		Unknown
10	B	Pain extremities	80/pos	<4/neg	<4/neg	<4/neg	-	-		2005
12	B	Exclusion NS	320/pos	8/pos	16/pos	<4/neg	-	-		2008
13	B	Symptoms	<80/neg	-	-	-	Balance disturbances, weakness extremities	3TC, ABC, NFV	> 1 month	Unknown
14	B	Symptoms	>2560/pos	4/pos	128/pos	<4/neg	Cognitive problems	-		2007
16	B	Symptoms	<80/neg	-	<4/neg	<4/neg	Progressive spinal cord lesion	-		2009
17	B	Symptoms	<80/neg	-	-	-	HIV encephalopathy	ZDV, RTV, ddI	> 10 years	1994
18	B	Symptoms	-	-	-	-	Seizures	3TC, ZDV, NVP	1 month	1999
19	B	Exclusion NS	>2560/pos	8/pos	8/pos	<4/neg	-	-		2007
20	B	Exclusion NS	>2560/pos	32/pos	8/pos	<4neg	-	-		2011
21	B	Symptoms	<80/neg	-	<4/neg	<4/neg	Radicular pain	ZDV, 3TC, TDF, LPV/r	1 year	2002
25	CRF12_BF	Symptoms	-	-	-	-	Headache, facialis paresis	-		2016
27	B	Symptoms	<80/neg	-	<4neg	-	Behavioral changes and aspecific white matter anomalies in Pons	Several, most recent: DRV/r, MVC, ETR	< 4 months	1994

Footnotes:

a Indication for lumbar puncture varied. Exclusion NS =Exclusion of neurosyphilis as part of standard clinical practice.

b Neurological diagnosis or symptoms. - = no neurological symptoms

c Time between cessation of ART and specimen collection
