# Supplementary material for: Characterization of HIV variants from paired Cerebrospinal fluid and Plasma samples in primary microglia and CD4+ T-cells
Source: J Neurovirol. 2024 May 7;30(4):380–92. doi: 10.1007/s13365-024-01207-w (PMC11512886; doi:10.1007/s13365-024-01207-w)
Supplement: Supplementary file 2 — Supplementary file2 (PDF 80 KB) [file 13365_2024_1207_MOESM2_ESM.pdf]

Supplementary Table 2: PCR primers sets for the amplification of HIV V3 and Env gene

HIV gene	PCR	Primers	Sequence 5' - 3'
V3	RT	V3-fw1	TATCCTTTGARCCAATTCCCAT
		V3-fw2	CAGTAGAAAAATTCCCCTCCACAA
	Nested	V3-rv3	AATTCCCCTCCACAATTAAAASTGTG
		V3-rv4	ACAGTACAATGTACACATGGAATTA
Env	RT	Oevif- 1forw	GGTCAGGGAGTCTCCATAGAATGGAGG
		HIV-R-end-rev1	GCACACAACGCGTGAAGCACTCAAGGCAAGCTTTATTGAGGC
	Nested	gp160fw-1	ACCAATAGTAGCAATAGTAGCATTAGTAGTAGCAGCAATAATAGCAATAGTTGTGTGG
		gp160fw-2	ACCAATAGTAGCAATAGTAGCATTAGTAGTAGCAGCAATAATAGCAATAGTTGTATGG
		gp160rv-3	GGAGTTCATGCTCAGCTCGTCTCATTCTTTCCCTTATAGCATGCCACCC
		gp160rv-4	GGAGTTCATGCTCAGCTCGTCTCATTCTTTCCCTTATAGTAGGCCATCC
